# Doubled haploid production from Spanish onion (*Allium cepa* L.) germplasm: embryogenesis induction, plant regeneration and chromosome doubling

**DOI:** 10.3389/fpls.2015.00384

**Published:** 2015-05-29

**Authors:** Oreto Fayos, María P. Vallés, Ana Garcés-Claver, Cristina Mallor, Ana M. Castillo

**Affiliations:** ^1^Unidad de Hortofruticultura, Centro de Investigación y Tecnología Agroalimentaria de AragónZaragoza, Spain; ^2^Departamento de Genética y Producción Vegetal, Estación Experimental de Aula Dei, Consejo Superior de Investigaciones Científicas (EEAD-CSIC)Zaragoza, Spain

**Keywords:** onion, gynogenesis, Spanish germplasm, flower bud, embryogenesis, Eco2box, chromosome doubling

## Abstract

The use of doubled haploids in onion breeding is limited due to the low gynogenesis efficiency of this species. Gynogenesis capacity from Spanish germplasm, including the sweet cultivar Fuentes de Ebro, the highly pungent landrace BGHZ1354 and the two Valenciana type commercial varieties Recas and Rita, was evaluated and optimized in this study. The OH-1 population, characterized by a high gynogenesis induction, was used as control. Growing conditions of the donor plants were tested with a one-step protocol and field plants produced a slightly higher percentage of embryogenesis induction than growth chamber plants. A one-step protocol was compared with a two-step protocol for embryogenesis induction. Spanish germplasm produced a 2–3 times higher percentage of embryogenesis with the two-step protocol, Recas showing the highest percentage (2.09%) and Fuentes de Ebro the lowest (0.53%). These percentages were significantly lower than those from the OH-1 population, with an average of 15% independently of the protocol used. The effect of different containers on plant regeneration was tested using both protocols. The highest percentage of acclimated plants was obtained with the two-step protocol in combination with Eco2box (70%), whereas the lowest percentage was observed with glass tubes in the two protocols (20–23%). Different amiprofos-methyl (APM) treatments were applied to embryos for chromosome doubling. A similar number of doubled haploid plants were recovered with 25 or 50 μM APM in liquid medium. However, the application of 25 μM in solid medium for 24 h produced the highest number of doubled haploid plants. Somatic regeneration from flower buds of haploid and mixoploid plants proved to be a successful approach for chromosome doubling, since diploid plants were obtained from the four regenerated lines. In this study, doubled haploid plants were produced from the four Spanish cultivars, however further improvements are needed to increase their gynogenesis efficiency.

## Introduction

Onion (*Allium cepa* L.) is a valuable crop for food and medicinal purposes, ranking second after tomato in the list of vegetables cultivated worldwide, with production of over 90 million tons on 4.7 million ha (FAO, 2013). Onion is an important crop in Spain, which is the third largest producer in Europe, after Russia and Netherlands. The onion production in Spain is over 1.2 million tons, ranking second among crop vegetables. Onion is an allogamous species and therefore both open pollinated cultivars and hybrids are cultivated. Hybrids have many advantages, including higher productivity, genetic uniformity and seed production for commercial use (Campion et al., 1995; Foschi et al., 2009). Uniform highly inbred lines are needed for hybrid production, but they are difficult to obtain through conventional methods of plant breeding (between 10 and 12 years) due to severe inbreeding depression and their biennial cycle, (Jakše et al., 2010). Haploid onion plant production and subsequent chromosome doubling offers a time-saving approach to obtain pure inbred lines (Dunwell, 2010; Chen et al., 2011). Onion breeding programs based on DH are being conducted at different public institutions such as Cornell University (Hyde et al., 2012), Wisconsin University in collaboration with Ljubljana University (Duangjit et al., 2013), Texas A&M University (Walker et al., 2006), INTA (Dr. Galmarini, personal communication), the Agricultural University of Kraków in collaboration with private companies (Adamus, personal communication), and Pamukale University (Alan et al., 2014; Celebi-Toprak et al., 2015).

Of the different methods for *in vitro* onion haploid production, only gynogenesis has been reported to be successful. Haploid onion plants have been produced from ovules, ovaries or whole flower buds (Muren, 1989; Campion and Alloni, 1990; Keller, 1990; Campion et al., 1992; Bohanec et al., 1995; Geoffriau et al., 1997; Michalik et al., 2000). Of the three aforementioned *in vitro* techniques, ovule culture was the least efficient. Ovary or flower bud culture showed similar results concerning embryo induction, but flower bud culture was less laborious (Bohanec et al., 1995; Bohanec and Jakše, 1999).

The main bottlenecks of gynogenesis in onion are the low rates of embryogenesis induction, plant survival and chromosome doubling from most of the materials (Geoffriau et al., 1997). Several aspects, including genotype and growing conditions of donor plants, culture medium and chromosome doubling procedure, need to be considered to achieve successful rates of gynogenesis (Bohanec, 2009; Chen et al., 2011). Material genotype and genetic structure are the most important factors (Jakše et al., 2010). Thus, low rates of gynogenesis induction have been reported in open-pollinated populations (0–3%) (Geoffriau et al., 1997; Bohanec and Jakše, 1999). Nevertheless, higher rates were achieved in specific synthetic populations, hybrid F1s, and inbred lines (10–33%) (Geoffriau et al., 1997; Bohanec and Jakše, 1999; Michalik et al., 2000; Bohanec et al., 2003).

Temperature stress treatment of donor plants, inflorescences, flowers or isolated ovules can trigger the switch from the gametophytic to the sporophytic pathway in different species (for review see Chen et al., 2011). In onion, the growth of donor plants at low temperatures with high illumination increased embryogenesis percentages (Puddephat et al., 1999; Michalik et al., 2001). However, the application of temperature stress treatment to flower buds or pre-growth on starvation medium did not enhance the rate of gynogenesis (Bohanec, 1998).

The first studies on onion gynogenesis were performed with ovary or ovule culture. In most cases, a two-step protocol was used including a pre-culture of the flower buds before ovary or ovule isolation. In these reports the basal media B5 (Gamborg et al., 1968), MS (Murashige and Skoog, 1962) and BDS (Dunstan and Short, 1977) were used (Muren, 1989; Campion and Alloni, 1990; Keller, 1990; Campion et al., 1992). Afterwards, flower bud culture protocols were developed based at first on those used for ovary and ovule culture with some modifications of the culture media, including growth regulators (Bohanec et al., 1995; Martínez et al., 2000; Michalik et al., 2000), and later on a simplified one-step protocol, consisting of culturing the whole flower bud in an induction medium until the embryo stage (Bohanec and Jakše, 1999; Jakše and Bohanec, 2003).

A low rate of spontaneous chromosome doubling has been described in onion gynogenesis and is below 10% in most cases (Alan et al., 2003; Jakše et al., 2010). Therefore, anti-mitotic agents have been applied to different explants to increase this rate, including small bulbs (Campion et al., 1995), plantlets during micropropagation (Geoffriau et al., 1997; Alan et al., 2004, 2007), and embryos (Jakše and Bohanec, 2000; Grzebelus and Adamus, 2004). Embryos have several advantages over other explants, including shortening the time needed for plant acclimation and avoiding the step of excision and regrowth of the bulb and/or plant (Jakše and Bohanec, 2000). A comparison of colchicine, trifluralin, orizalin and amiprofos-methyl (APM) treatments showed that colchicine was the least efficient in chromosome doubling and trifluralin and orizalin resulted in higher hyperhydricity (Grzebelus and Adamus, 2004). Alan et al. (2007) compared different strategies for ploidy level manipulation in onion gynogenesis, reporting that somatic regeneration of spontaneous DH plants from flower buds of haploid and mixoploid plants was the most reliable. This strategy was applied later by Jakše et al. (2010).

The main objective of this study was to evaluate the gynogenesis capacity of Spanish onion germplasm using flower bud culture and to optimize the percentage of acclimated doubled haploid (DH) plants. Two gynogenesis induction protocols, previously described in the literature, different plant containers for plant regeneration, and APM treatments for chromosome doubling, were assayed. Somatic regeneration was also tested as an alternative approach for chromosome doubling of haploid (H) gynogenetic plants.

## Materials and methods

### Material

Research was carried out from 2012 to 2014 in experimental plots in a shade house located at 41°39′N latitude. In 2012, two Valenciana type commercial varieties Recas (Veronsa) and Rita (kindly provided by Ramiro Arnedo S.A.) were used, as well as a half-sib family from the breeding program carried out with the cultivar Fuentes de Ebro, a landrace known for its mild and sweet flavor (Mallor et al., 2011a; Mallor and Sales, 2012). Fuentes de Ebro has a high commercial value due to its differentiated quality, provided by the Protected Designation of Origin (PDO)^1^ label, according to Regulation (EEC) 1146/2013 of the European Union. In 2013, the cultivars Fuentes de Ebro, Recas, and BGHZ1354, and the population OH-1 were used. BGHZ1354 is a landrace provided by the Vegetable Germplasm Bank of Zaragoza (BGHZ, Zaragoza, Spain) characterized by a high level of pungency (Mallor et al., 2011b). OH-1 is a synthetic population specially designed for high gynogenesis induction, obtained with inbred lines B2923B and B0223B (Havey and Bohanec, 2007).

### Growing conditions of donor plants

In February 2011, seeds of donor plants (Fuentes de Ebro, Rita and Recas were sown in polystyrene trays in a greenhouse with a substrate mixture of peat (50%), coconut (30%), sand (20%), and N:P:K (14:16:18) with micronutrient fertilizer (Projar S.A., Valencia, Spain). In April, the plantlets were transplanted directly to soil in the field under natural conditions and bulbs were collected for repose in September. In order to obtain flower heads, the bulbs were planted in November in plastic pots (4 l) and grown in the field under natural conditions. One month before bolting, some bulbs were transferred to a growth chamber exposed to 16 h photoperiod at a continuous temperature of 15–18°C and an illumination of 300 μmol m^−2^ s^−1^provided by Philips Master SON-T, PIA Hg Free 150 W and Phillips Master TL_D 58 W/865. The rest of the bulbs were kept in the field under natural conditions.

In February 2012, seeds of donor plants from Fuentes de Ebro, BGHZ1354, Recas, and OH-1 were sown, grown and harvested as described previously. In November 2012, the bulbs were transplanted directly to soil in the field under natural conditions for inflorescence development in 2013. Whole umbels were harvested from mid-May to the end of June.

### Sterilization

**Figure 1:**
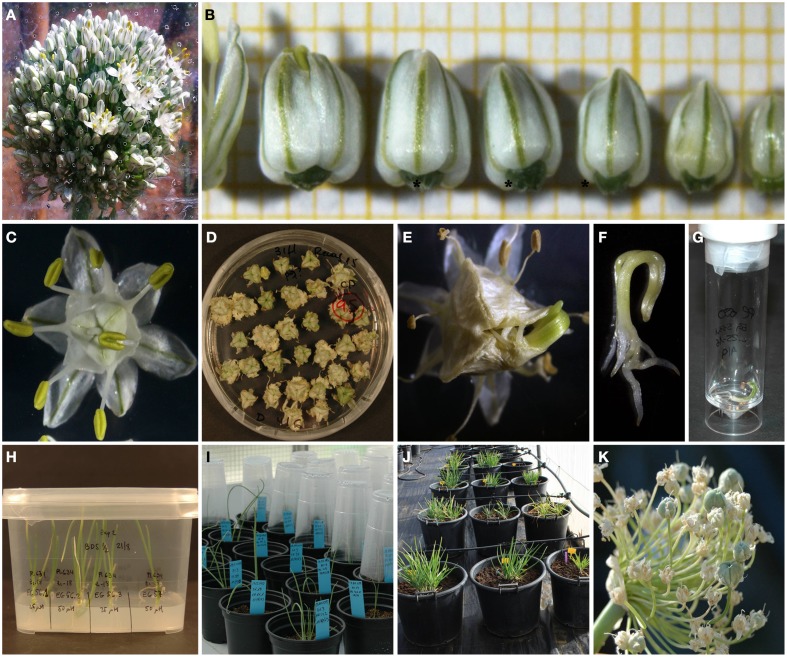
**Production of doubled haploid onion plants by gynogenesis. (A)** Onion umbels at the time of harvest. **(B)** Optimal stage of flower bud development for culture (3.5–4.5 mm length, flowers tagged with an asterisk). **(C)** Flower after 7 days of culture. **(D)** Flowers from cultivar Recas after 45 days of culture. **(E)** Gynogenetic embryo from Fuentes de Ebro emerging from the ovary at 90 days of culture. **(F)** Isolated embryo from Rita. **(G)** Onion embryo treated with APM in liquid medium. **(H)** OH-1 plant regeneration in Eco2box. **(I)** Plants during acclimation in greenhouse. **(J)** Bulb formation in a shade house. **(K)** Seed production from Rita.

The whole umbel was harvested when 30% of the flowers were at three to 4 days before anthesis (Figure 1A). Flowers of 3.5–4.5 mm in length were selected (Figure 1B) from each umbel and sterilized in ethanol 70% for 2 min and 16.5 g l^−1^ dichloroisocyanuric acid disodium salt with two drops of Tween 80 for 10–12 min and followed by 4–5 rinsed with sterile distilled water.

### Culture media

Two different protocols were assayed for gynogenesis induction. In 2012, the protocol described by Jakše and Bohanec (2003) (Protocol A) was followed. Flower buds were cultured on an induction medium consisting of BDS medium (Dunstan and Short, 1977) with some modifications (Supplemental Table S1) and were kept in the same medium until embryo production (one-step protocol). In 2013, a two-step protocol described by Michalik et al. (2000) (Protocol B) was also used. In this protocol, flower buds were plated on A_1_ medium (Muren, 1989) for 1 month and later transferred to R_1_ medium (Michalik et al., 2000) (Supplemental Table S1) until embryo production. For protocol comparison, flower buds from the same umbel were randomly distributed in each protocol. Thirty flowers were inoculated in each 90 mm Petri dish.

### Chromosome doubling

Embryos were used as explants for chromosome doubling. The duplication agent amiprofos-methyl (APM) was applied in liquid or solid elongation medium and consisted of BDS (x1/2) with 15 g l^−1^ glucose (Supplemental Table S1). In 2012, 25 μM APM was applied for 24–48 h in liquid medium. In 2013, concentrations of 25 and 50 μM APM were applied for 24 h in liquid elongation medium in a first experiment. In a second experiment, APM (25 μM) was applied in liquid medium for 24 h or in solid medium for 24 or 72 h. Good quality embryos from the “OH-1” population were randomly distributed in different treatments. APM treatment was performed in the dark and afterwards embryos were rinsed with liquid elongation medium.

### Plant regeneration, acclimation and bulb and seed production

In 2013, two experiments were performed. In the first, glass tubes were sealed with plastic or cellulose plugs (Steristophen Typ 20, Carl Roth GmBH+Co). In the second, glass tubes with plastic plugs were compared with Magenta boxes and polypropylene boxes with an aeration filter (Eco2box-green filter-Model 80MM H, Duchefa). Two to three plants were plated in each Magenta box and 4–6 plants in the Eco2box. Good quality embryos from the OH-1 population were randomly distributed in different containers.

In 2012, APM treated embryos were cultured in solid elongation medium in individual glass tubes with plastic plugs (150 × 25 mm). In 2013, glass tubes with plastic glass and Eco2boxes were used for plant regeneration from Spanish germplasm.

### Culture conditions

Flower buds and plantlets were incubated in a growth chamber at 24°C with 16 h photoperiod and 100 μmol m^−2^ s^−1^ provided by Phillips Master TLD Super 80 58 W/840 and OSRAM 30 W/2700 K.

After 6–10 weeks in elongation medium, rooted, healthy plants were transferred to 110 mm Ø pots containing soil (3:2 peat:vermiculite), covered with a plastic glass for 10 days and planted in a greenhouse. Plants were watered with Hoagland nutrient solution (Hoagland and Arnon, 1950) for the first month. For bulb and seed production, all H, mixoploid and DH plants were transplanted to 50 l plastic pots in the shade house with the same substrate mixture that had been used for donor plant growing.

### Somatic regeneration

Flower buds from H and mixoploid lines obtained in 2013 that bolted in July 2014 were harvested at the same stage as for gynogenesis induction, and cultured following the protocol described by Luthar and Bohanec (1999). Flower buds were inoculated in induction medium (I) for 7–8 days and then transferred to differentiation medium (R_2_) (Supplemental Table S1). Shoot clumps were transferred to Magenta boxes containing solid elongation medium.

### Ploidy analysis

Ploidy was estimated by flow cytometry from acclimated plants. Young leaves were chopped in 2 ml Cystain UV ploidy solution (Partec) and filtered through a 30 μm nylon filter. Samples were analyzed in a PAS cytometer (Partec). Leaves from young seedlings were used as control.

### Statistical analysis

The following variables were calculated: percentage of gynogenesis induction (number of embryos or calli/100 flowers), percentage of embryogenesis induction (number of embryos/100 flowers), percentage of acclimated plants (number of acclimated plants/100 embryos), percentage of hyperhydricity (number of hyperhydrated plants/100 embryos), percentage of H, mixoploid (n/2n), DH and tetraploid as the number of plants/100 acclimated plants. All experiments were established in a completely randomized design. Percentages of gynogenesis induction, embryogenesis induction, acclimated plants, hyperhydricity, ploidy levels and globular structures were analyzed using the Chi Square test by the FREQ procedure.

## Results

### Effect of donor plant growing conditions on gynogenesis induction and plant production

Bulbs from Fuentes de Ebro, Recas, and Rita were grown in the field and in a growth chamber. A total number of 9934 flower buds from the three cultivars were grown in 2012 following the protocol described by Jakše and Bohanec (2003) (Protocol A) (Table 1). Flowers opened after 3–6 days of culture (Figure 1C), stayed green during the first month of culture and progressively turned yellow as the culture progressed. Some flowers from Recas and Fuentes de Ebro developed small calli on the flower base after 30–40 days of culture (Figure 1D). Gynogenetic embryos emerged directly from the ovules rupturing the ovary wall after 70–150 days of culture (Figure 1E). The percentage of gynogenesis induction and the percentage of embryogenesis were quite similar, as calli were rarely formed (Table 1). Both variables depended on the cultivar and the growing conditions of the donor plants. The highest percentage of embryogenesis induction was produced from Recas, followed by Rita and Fuentes de Ebro. No statistically significant differences were found in percentages of gynogenesis and embryogenesis induction between the two growing conditions for all cultivars. However, field plants of Fuentes de Ebro and Rita rendered an 80% higher percentage of embryogenesis than growth chamber plants.

**Table 1:** **Spanish germplasm gynogenesis following the protocol described by Jakše and Bohanec (2003) (Protocol A) in 2012**.

**Cultivar**	**Growing conditions**	**Flowers**	**Gynogenesis induction**		**Embryogenesis induction**		**Acclimated plants**	
			**Calli + Embryos**	**Calli+Embryos/Flowers (%)**	**Embryos**	**Embryos/Flowers (%)**	**Plants Plants**	**Plants/Embryos (%)**
Fuentes de Ebro	F^*^	1206	9	0.75a^***^	8	0.66a^***^	1	12.5a^***^
	GC^**^	1688	7	0.41a	6	0.36a	0	0.0b
Recas	F	1998	30	1.50a	28	1.40a	0	0.0b
	GC	1062	16	1.51a	14	1.32a	1	7.1a
Rita	F	2966	49	1.65a	42	1.42a	2	4.8b
	GC	1014	11	1.08a	8	0.79a	2	25.0a

* F, Field;

** GC, Growth Chamber;

*** *Values followed by the same letter within the cultivar are not significantly different (P < 0.05)*.

Well-developed gynogenetic embryos (Figure 1F) were treated with APM in elongation medium (Figure 1G). Then embryos were transferred to a solid elongation medium for plant development (Figure 1H), and afterwards the plants were transplanted to plastic pots into the greenhouse for acclimation (Figure 1I) and bulb and seed production (Figures 1J,K). A high proportion of embryos developed abnormal or hyperhydrated plants, up to 50% in Fuentes de Ebro and 21% in Recas (data not shown), leading to a low percentage of acclimated plants. A higher percentage of acclimated plants was obtained from field plants in Fuentes de Ebro (12.5%) and from growth chamber plants in Recas and Rita (7.14 and 25%, respectively). Two plants obtained from Fuentes de Ebro and Recas were DH and H, respectively, and those from Rita were one DH and two mixoploids (n/2n) (data not shown).

### Effect of culture protocol on gynogenesis induction and plant production

Since low percentages of embryogenesis induction and acclimated plants were obtained in 2012, the two-step protocol described by Michalik et al. (2000) (protocol B) was compared with protocol A. The OH-1 population was included in order to determine whether the frequencies of gynogenesis induction obtained were within the predicted range. The gynogenesis capacity of landrace BGHZ1354, was also evaluated. Plants were grown in the field, since in 2012 the percentage of embryogenesis induction was slightly higher in 2 out of the 3 cultivars in this growing condition, and also the number of plants that can be grown is not limited by the growth chamber capacity. A total number of 10,767 flowers from Fuentes de Ebro, Recas, BGHZ1354, and OH-1 was cultured (Table 2). Different rates of embryogenesis induction were obtained with the two protocols, depending on the material. Spanish germplasm produced 2–3 times higher percentages of embryogenesis with protocol B, Recas showing the highest percentage (2.09%), followed by BGHZ1354 with 1.28% and Fuentes de Ebro with 0.53%. Recas and BGHZ1354 had statistically significant differences between protocols but not the lowest responding cultivar. The percentage of embryogenesis from Spanish germplasm was significantly lower than those obtained from the OH-1 population, where no differences for percentage of embryogenesis between protocols were observed (around 15%).

**Table 2:** **Spanish germplasm gynogenesis following protocol described by Jakše and Bohanec (2003) (Protocol A) and described by Michalik et al. (2000) (Protocol B) and the population OH-1 (Havey and Bohanec, 2007)**.

**Cultivar**	**Protocol**	**Flowers**	**Embryogenesis induction**		**Acclimated plants**		**Ploidy level of acclimated plants**		
			**Embryos**	**Embryos/Flowers (%)**	**Plants**	**Plants/Embryos (%)**	**n**	**n/2n**	**2n**
Fuentes de Ebro	A	1125	3	0.27a^*^	1	33.3a^*^	1	–	–
	B	1127	6	0.53a	2	33.3a	1	–	1
Recas	A	1149	10	0.87b	1	10.0b	1	–	–
	B	1149	24	2.09a	8	33.3a	3	3	1
BGHZ1354	A	1486	6	0.40b	1	16.7b	–	1	–
	B	1481	19	1.28a	6	31.5a	4	1	1
OH-1	A	1624	244	15.00a	84	34.5a	ND	ND	ND
	B	1626	250	15.42a	118	47.2a	ND	ND	ND

* *Values followed by the same letter within the cultivar are not significantly different (P < 0.05)*.

The percentage of acclimated plants from both protocols also varied depending on the cultivar (Table 2). In BGHZ1354 and Recas, protocol B gave rise to a 2- and 3-fold increase, respectively, in comparison to protocol A. However, no differences in percentage of acclimated plants between protocols were observed in Fuentes de Ebro. Although the percentage of acclimated plants from OH-1 rose from 34 to 47% with protocol B, these differences were not statistically significant.

Furthermore, differences in percentages of embryogenesis induction and plant acclimation were also observed between plants from the same cultivar. Only 2 out of 9 plants from Fuentes de Ebro produced embryos, the highest percentage of embryogenesis being 3.3% with protocol B and 2.0% with protocol A. In OH-1, this percentage varied from 0 up to 93% with protocol B and from 0 to 76% with protocol A (data not shown).

### Effect of the container during plant regeneration

In order to reduce plant hyperhydricity, two experiments were performed to assay containers with different levels of ventilation, using OH-1 embryos obtained with both induction protocols (Table 3). In the first, glass tubes sealed with plastic and cellulose plugs were assayed. No statistically significant differences in percentages of hyperhydricity and acclimated plants were observed between plastic and cellulose plugs, independently of the protocol used. The percentage of hyperhydricity almost doubled in plants obtained with protocol B than with protocol A.

**Table 3:** **Effect of plant container during plant regeneration in gynogenesis of OH-1 population**.

**Experiment**	**Protocol**	**Treatment**	**Embryos**	**Hyperhydricity**		**Acclimated plants**	
				**Plants**	**Plants/Embryos (%)**	**Plants**	**Plants/Embryos (%)**
Glass tube plugs	A	Plastic	40	5	12.5a^*^	12	30.0a^*^
		Cellulose	43	5	11.6a	16	37.2a
	B	Plastic	30	7	23.3 a	13	43.3a
		Cellulose	24	5	20.8a	8	33.3a
Container	A	Glass+plastic	35	6	17.1a	7	20.0b
		Eco2box	28	3	10.7a	7	25.0ab
		Magenta	16	3	18.8a	6	37.5a
	B	Glass+plastic	26	9	34.6b	6	23.1c
		Eco2box	24	1	4.2a	17	70.8a
		Magenta	23	8	34.8b	12	52.2b

* *Values followed by the same letter within the cultivar are not significantly different (P < 0.05)*.

In the second experiment, different types of plant containers were compared; glass tube with plastic plug, Eco2box and Magenta box (Table 3). An interaction between container and protocol was observed for percentages of hyperhydricity and acclimated plants. The lowest percentage of hyperhydricity (4.17%) and the highest percentage of acclimated plants (up to 70%) were obtained with Eco2box and protocol B. In protocol A, no significant differences for hyperhydricity percentages were observed between the different types of container, but Magenta boxes produced the highest percentage of acclimated plants (37.5%). In both protocols glass tubes rendered the lowest percentage of acclimated plants (20–23%). As in the previous experiment, protocol B rendered higher percentages of hyperhydricity and acclimated plants than protocol A, except when Eco2 boxes were used.

### Effect of APM application on chromosome doubling

Two concentrations of APM were applied in liquid medium to OH-1 embryos to optimize the percentage of chromosome doubling (Table 4). No significant differences in the percentages of acclimated plants and diploid plants were observed between the two concentrations. However, the percentage of acclimated plants decreased from 55 to 37% when the concentration of APM was raised from 25 to 50 μM, and the percentage of DH plants increased up to 38%. As a consequence, a similar number of DH plants were produced with both concentrations. Two plants with higher ploidy levels (4n) were also obtained with the lower concentration of APM.

**Table 4:** **Effect of APM application on chromosome doubling in OH-1 embryos**.

**Experiment**	**Treatment**	**Embryos**	**Acclimated plants**		**Ploidy level of acclimated plants**			
			**Plants**	**Plants/Embryos (%)**	**n (%)**	**n/2n (%)**	**2n (%)**	**4n (%)**
APM concentration	25 μM APM	69	38	55.1a^*^	17 (44.7a^*^)	8 (21.1a^*^)	11 (28.9a^*^)	2 (5.3^*^)
	50 μM APM	70	26	37.1 a	9 (34.6a)	6 (23.1a)	10 (38.5a)	0 (0.0)
Medium support	25 μM APM liq (24 h).	44	12	27.2a	6 (50.0a)	3 (25.0b)	3 (25.0ab)	0 (0.0)
Aplication time	25 μM APM sol (24 h)	42	17	40.5a	10 (58.8a)	1 (5.9c)	6 (35.3a)	0 (0.0)
	25 μM APM sol (72 h)	41	14	34.2a	3 (21.4b)	8 (57.1a)	2 (14.3b)	1 (7.1)

* *Values followed by the same letter within the cultivar are not significantly different (P < 0.05)*.

In a second experiment, APM application was compared in solid and liquid media. No significant differences in the percentage of acclimated plants were observed between the application of APM for 24 h in solid and liquid media, but there was a slight increase in the solid medium (from 27 to 40%). APM application in solid medium for 72 h slightly decreased plant survival and produced the lowest percentage of haploid and diploid plants, but up to 57% of mixoploid plants. The highest percentage (35%) and number of DH plants was reached when 25 μM APM was applied in solid medium for 24 h.

### Plant production from Spanish germplasm in 2013

Plant production for the onion breeding program was carried out in parallel with experiments described above for the optimization of DH plant production. Protocol A was followed and embryos were treated with 25 μM APM in liquid medium for 24 h as performed in 2012, but embryos were transferred to Eco2boxes or glass tubes for plant elongation. A total number of 15,064 flower buds from plants grown in the field from Fuentes de Ebro, Recas, BGHZ1354 and OH-1 were inoculated (Table 5). Great differences were observed between cultivars in the percentage of embryogenesis induction. As expected, OH-1 produced the highest percentage of embryogenesis (18.2%), obtaining a significantly lower rate with the Spanish germplasm. BGHZ1354 showed the highest percentage of embryogenesis (1.05%), and Fuentes de Ebro the lowest (0.52%). These percentages are higher than those obtained with a smaller number of flowers in the experiment to compare protocols (Tables 2, 5). However, similar percentages of embryogenesis were produced from the Recas cultivar.

**Table 5:** **Gynogenesis induction from Spanish germplasm in 2013 following Protocol (A) from Jakše and Bohanec (2003) with some modifications**.

**Cultivar**	**Flowers**	**Embryogenesis induction**		**Acclimated plants**		**Ploidy level of acclimated plants**		
		**Embryos**	**Embryos/Flowers (%)**	**Plants**	**Plants/Embryos (%)**	**n (%)**	**n/2n (%)**	**2n (%)**
Fuentes de Ebro	6998	18	0.25d^*^	3	16.67b^*^	2 (66.7a^*^)	0 (0.0b^*^)	1 (33.3b^*^)
Recas	3227	24	0.74bc	4	16.67b	2 (50.0b)	0 (0.0b)	2 (50.0a)
BGHZ1354	3819	40	1.05b	13	32.50ab	5 (50.0b)	3 (30.0a)	2 (20.0c)
OH-1	4533	826	18.22a	302	36.60 a	–	–	–

* *Values followed by the same letter within the cultivar are not significantly different (P < 0.05)*.

As for the Spanish germplasm, BGHZ1354 showed the highest percentage of acclimated plants (32.5%), with close rates to those obtained from OH-1. Fuentes de Ebro and Recas reached almost 17%. These percentages were significantly higher than those obtained in 2012 (Tables 1, 5). Different rates of DH plants were obtained from each cultivar: 50, 33, and 20% from Recas, Fuentes de Ebro, and BGHZ1354, respectively. A level of 30% mixoploid (n/2n) plants was obtained from BHGZ1354 (Table 5).

### Chromosome doubling by flower bud somatic regeneration

Somatic regeneration from flower buds following the protocol described by Luthar and Bohanec (1999) was performed as an alternative approach for chromosome doubling from haploid and mixoploid (n/2n) plants. Five out of the 33 acclimated plants from Spanish germplasm (Tables 2, 5), and 2 out of the 21 plants from the OH-1 population flowered in July 2014 and were used for somatic regeneration (Table 6). After 3–4 weeks of culture, globular embryogenic structures developed on the bases of the flower buds. Large differences in percentages of embryogenic structures and acclimated plants were observed among cultivars and lines. The haploid line from Fuentes de Ebro produced a medium percentage of embryogenic structures (33%), and a low rate of acclimated plants (2.5%). Of the lines from BGHZ1354, a high percentage of embryogenesis was observed from lines 13.P.045 (72%) and 13.P.074 (53%). The other lines from BGHZ1354 and the 2 lines from OH-1 never or rarely developed embryogenic structures. Shoots were regenerated from lines 13.P.041, 13.P.045, and 13.P.074 that had a high percentage of globular structures, and even from the low embryogenic line 13.P.042. No acclimated plants were produced from OH-1.

**Table 6:** **Chromosome doubling of gynogenetic haploid and mixoploid plants by flower bud somatic regeneration**.

**Origin**	**Gynogenic line**	**Initial ploidy level**	**Flowers**	**Globular structures (%)**	**Acclimated plants**		**ploidy level of acclimated plants**			
					**Plants**	**Plants/100 Flowers**	**n (%)**	**2n (%)**	**2n/4n (%)**	**4n (%)**
Fuentes de Ebro-17	13.P.041	N	122	32.8c^*^	2	1.6bc^*^	1 (50.0a^*^)	1 (50.0c^*^)	0	0
BHGZ1354-16	13.P.045	n	255	71.8a	16	6.3b	7 (43.8a)	8 (50.0c)	1 (6.0a^*^)	0
BHGZ1354-1	13.P.042	n	134	3.0d	2	1.5bc	0	2 (100.0a)	0	0
BHGZ1354-14	13.P.213	n/2n	182	0.0d	0	0.0c	0	0	0	0
BHGZ1354-17	13.P.074	n/2n	309	52.8b	50	16.2a	0	44 (88.0b)	3 (6.0a)	3 (6.0)
OH-1-8	13.E2.034	n	55	0.0d	0	0.0c	0	0	0	0
OH-1-8	13.E2.040	n	160	2.5d	0	0.0c	0	0	0	0

* *Values followed by the same letter within lines are not significantly different (P < 0.05)*.

Differences in the ploidy level of somatic regenerated plants were observed between H lines. The two plants from 13.P.042 were diploid, whereas similar percentages of H and diploid plants were obtained from 13.P.045. The mixoploid line that produced embryogenic structures rendered up to 88% of diploid plants. Mixoploid (2n/4n) and tetraploid plants were also produced from H and mixoploid plants.

## Discussion

The availability of protocols for onion DH production represents a unique opportunity to have completely homozygous and stable inbred lines. Gynogenesis capacity from Spanish germplasm has been evaluated and different protocols have been assayed to increase the number of acclimated plants, in order to introduce pure inbred lines in an onion breeding program, to improve bulb size, uniformity and storability (Mallor and Sales, 2012). Spain is the third largest producer in Europe with over 1.2 million tons. We should remark that the cultivar “Fuentes de Ebro” used in this study has a high commercial value due to its differentiated quality. Genetic factors, including cultivar, donor plant genotype, geographic origin and genetic structure are thought to be the most important for the success of gynogenesis induction (Campion and Alloni, 1990; Geoffriau et al., 1997; Bohanec and Jakše, 1999; Michalik et al., 2000; Chen et al., 2011). In Spanish germplasm, cultivar and plant genotype also has a strong effect on gynogenesis induction. The two Valencia-type cultivars Rita and Recas showed the highest embryogenesis percentage (0.87–2.09%), followed by the highly pungent landrace BGHZ1354 (0.40–1.28%), and finally the sweet cultivar Fuentes de Ebro (0.27–0.75%). A similar percentage of embryogenesis induction was described in the unique Spanish cultivar Morada de Amposta evaluated previously (2.1%) and in 5 Portuguese cultivars (average 0.98%) (Bohanec and Jakše, 1999). However, gynogenesis capacity of Spanish germplasm used in this study was lower than that from the American OH-1 population, and other American inbred and F1s reported by Bohanec and Jakše (1999). It has also been reported that American materials were on average 5 times more responsive than European cultivars (Bohanec et al., 2003), and northern European cultivars were more responsive than southern- and eastern European material (Geoffriau et al., 1997; Bohanec and Jakše, 1999). On the other hand, open pollinated cultivars, such as the ones used in this study, had shown a lower percentage of embryogenesis than inbred lines or F1s (Geoffriau et al., 1997; Bohanec and Jakše, 1999). Deleterious genes regulating vegetative growth are responsible for hampering gynogenic embryo development and plant regeneration and are eliminated during inbreeding (Geoffriau et al., 1997; Hyde et al., 2012).

A great variation in gynogenesis induction was also reported even between plants from the same inbred line. By way of example, B2923B showed a percentage of embryogenesis from 2.1 to 54.7% (Bohanec et al., 2003). Similar results were obtained in this study among individual plants from the same cultivar or population, since each plant was a different genotype. In this study, plant response from the synthetic population OH-1, characterized by its high embryogenesis induction and obtained from inbreeds B2923B and B0223B (Prof Havey, University of Wisconsin), varied from 0 to 93%. As for the Spanish germplasm, the highest variation, from 0 to 11%, was found in Recas.

The environmental conditions under which the donor plants are grown are also a key factor influencing embryogenesis induction (Campion et al., 1992; Chen et al., 2011). Onion plants in the growth chamber during bolting at 14–15 °C produced higher percentages of embryogenesis than plants in the greenhouse (13–25°C) (Puddephat et al., 1999) or plants in growth chamber at 4°C or 18°C or in the field (Michalik et al., 2001). In this study, no statistically significant differences were obtained between the two growing conditions, probably due to the low number of embryos produced. However, Fuentes de Ebro and Rita plants grown in the field rendered a slightly higher percentage of embryogenesis than those in the growth chamber at 15–18°C.

The differences in the percentage of gynogenesis induction from plants grown under natural conditions observed between years could be due to environmental, climatic or physiological conditions, as they were grown in pots or in soil under natural conditions, as well as genotype effects. A variation in the percentage of gynogenesis induction between the same bulbs over 2 years (23.2–32.9%) has been described in an onion inbred line (Bohanec et al., 2003).

Gynogenesis response of onion material depended on the culture media used (Campion et al., 1992; Jakše et al., 1996; Michalik et al., 2000). Thus, local cultivars from Argentina produced more haploid plants in media containing polyamines than without growth regulators (Martínez et al., 2000). In order to improve the percentage of embryogenesis obtained with our material in 2012 with the one-step protocol (protocol A) described by Jakše and Bohanec (2003), a two-step protocol (protocol B), that had been proved to induce gynogenesis efficiently in Polish cultivars (Michalik et al., 2000), was also assayed. A real comparison of the two protocols was possible since the same cultivars have been used. The low-responding Spanish material produced 2–3 times higher percentages of embryogenesis and plant acclimation with the two-step protocol described by Michalik et al. (2000). No significant differences were obtained in Fuentes de Ebro, probably due to the low number of embryos produced. The high gynogenetic population OH-1 produced similar percentages of embryogenesis, an average of 15%, independently of the protocol used. It had been reported previously that inbred lines B2923B and B0223B, used for the creation of the OH-1 synthetic population, reached 18-33% of gynogenesis induction (Bohanec et al., 2003; Jakše and Bohanec, 2003). Therefore, our results are comparable to those described by these authors.

The protocols differed in culture medium composition, vitamins, amount of ammonium and growth regulators. Regarding the latter, 2iP and NAA in protocol B, instead of 2,4-D and BA (protocol A) could account for the higher gynogenesis efficiency. Higher percentages of embryogenesis were also reported with Polish onion cultivars, with media containing 2iP and NAA (Michalik et al., 2000). The effect of growth regulators on plant acclimation confirms the results described by Bohanec et al. (1995). Hence, protocol B will be adopted for the development of onion haploids in our laboratory.

The presence of an unbalanced gaseous environment in the culture container could lead to plant hyperhydricity (Kozai and Smith, 1995; Lai et al., 2005). The use of different containers and/or container closure could cause different rates of gas exchange. In this study, a high percentage of hyperhydricity was observed during the first year, especially in Fuentes de Ebro (up to 50%) and Recas (21%). Different types of plug for glass tubes and containers were tested to decrease hyperhydricity and even though cellulose plugs were expected to reduce hyperhydricity and favor a better gas exchange than plastic plugs, a similar percentage of hyperhydricity was obtained with both types of plug in this study. Nevertheless, hyperhydricity was reduced to 4% and the percentage of acclimated plants was increased up to 70% with Eco2box in protocol B. This could be due to the presence of an aeration filter in the lid, favoring gas exchange. Our results agree with those reported in other crops in which hyperhydricity was reduced or even completely eliminated in ventilated cultures (Zobayed et al., 1999; Lai et al., 2005; Casanova et al., 2008; Ivanova and Van Staden, 2010). As far as we know, Eco2boxes have not been used previously for plant regeneration in onion. The differences observed from Spanish germplasm in the percentage of acclimated plants between years could be due to the use of both Eco2boxes and glass tubes for plant regeneration in the second year, but exclusively glass tubes in the first year. Other important factors associated with hyperhydricity are the amount of ammonium and the type of cytokinin in the medium (Ivanova and Van Staden, 2008). Since ammonium was only used in the first month of culture in the two-step protocol, the higher percentage of hyperhydricity produced with this protocol could be related to the use of the cytokinin 2iP instead of BA. However, BA has been reported to produce higher rates of hyperhydricity than other cytokinins such as kinetin, zeatin or thidiazuron (Chukwujekwu et al., 2002; Chen et al., 2006; Ivanova and Van Staden, 2011).

Several duplication agents and different strategies have been used for chromosome doubling of onion haploid embryos/plants (Campion et al., 1995; Alan et al., 2004; Grzebelus and Adamus, 2004). In general, the optimal concentration of the duplication agent and the duration of treatments must always be determined in relation to the percentage of doubled plants and the percentage of plant survival (Bohanec, 2009; Castillo et al., 2009). Although a relatively low number of embryos was used for each treatment, an increase in the concentration of APM from 25 to 50 μM in a liquid medium significantly increased the percentage of chromosome doubling. However, the same number of DH plants was obtained in both concentrations, since the high concentration reduced the percentage of plant survival. Similar results were reported previously in onion by Jakše et al. (2003). A higher percentage of diploid plants were produced when duplication agents were incorporated in liquid rather than in solid media (Jakše et al., 2003; Alan et al., 2004). However, in our study APM in a solid medium produced a higher survival rate (40%) and a higher percentage of doubling (35%) than in a liquid medium, resulting in 14% of DH/embryos treated. This percentage was higher than the 4% obtained by Jakše et al. (2003) with the same concentration of APM and application time. The differences between these two studies could be due to the use of different genotypes, since a genotype effect has been reported (Alan et al., 2004). In this study, increasing the time of application from 24 to 72 h in a solid medium significantly increased the percentage of mixoploid (n+2n) plants up to 57%, confirming the results reported by Jakše et al. (2003).

An alternative approach for chromosome doubling in onion is *in vitro* adventitious somatic regeneration (Alan et al., 2007; Jakše et al., 2010). This method has two advantages over the application of antimitotic agents: no potentially damaging chemicals are used and a higher percentage of doubling efficiency can be achieved (Alan et al., 2007; Jakše et al., 2010). When the protocol described by Luthar and Bohanec (1999) was applied, a large variation in somatic regeneration response was observed between different germplasms. The landrace BGHZ1354 showed the highest percentage of globular embryogenic structures (up to 72%), followed by “Fuentes de Ebro.” No correlation between somatic and gynogenetic embryogenesis was observed, since DH lines 13.E2.034 and 13.E2.040 derived from OH-1-8 plant produced a low percentage of embryogenic structures (0–2.5%), whereas OH-1-8 showed the highest percentage of embryogenesis induction (76–98%). Similar results were observed from line 13.P.042, indicating that genetic control for somatic regeneration and gynogenesis induction are inherited independently.

In the first study using this strategy for chromosome doubling 60% of the somatic plants regenerated from haploid flowers were spontaneously doubled (Alan et al., 2007). Jakše et al. (2010) produced diploid plants from 83 and 100% of regenerated haploid and mixoploid lines, respectively, and the average percentages of diploid plants among the regenerants was 55.8% from mixoploid lines and 9.7% from haploid lines. In this study, 4 out of the 7 lines that bolted the first year were regenerated, and diploid plants were produced from all of them (100%). The percentage of spontaneous doubling was 88% from the mixoploid line and between 47 and 100% from the haploid lines. Our results confirm that somatic regeneration is an alternative approach for chromosome doubling in onion. As described by Alan et al. (2007), the use of different strategies offers an integrated approach to recover diploid onion gynogenetic plants. In this study, both the application of APM to embryos, and diploidization of haploids and mixoploid lines by somatic regeneration were used successfully.

The gynogenesis capacity of Spanish germplasm has been evaluated. This germplam showed a low capacity for embryogenesis induction, the sweet cultivar Fuentes de Ebro being the most recalcitrant. For the first time comparison of two protocols has been addressed, the protocol described by Michalik et al. (2000) being more efficient for embryogenesis induction and acclimated plants than the one described by Jakše and Bohanec (2003). A new generation of tissue culture vessel, the Eco2box container, significantly reduced the percentage of hyperhydricity leading to a higher percentage of plant survival. Two strategies for chromosome doubling, the application of 25 μM APM in solid medium for 24 h and somatic regeneration from flower buds of haploid and mixoploid plants, were successfully used. Although gynogenetic plants were obtained from all the Spanish cultivars, further improvements of the protocol using different strategies should be performed in order to produce a high number of DH plants that could be used efficiently in onion breeding programs.

### Conflict of interest statement

The authors declare that the research was conducted in the absence of any commercial or financial relationships that could be construed as a potential conflict of interest.

## Footnotes

^1^Commission Implementing Regulation (EU) No. 1146/2013 of 5 November 2013 on entering a name in the register of protected designations of origin and protected geographical indications [Cebolla Fuentes de Ebro (PDO)].

## Supplementary material

The Supplementary Material for this article can be found onlineat: http://journal.frontiersin.org/article/10.3389/fpls.2015.00384/abstract
