# Supplementary material for: Doubled haploid production from Spanish onion (Allium cepa L.) germplasm: embryogenesis induction, plant regeneration and chromosome doubling
Source: Front Plant Sci. 2015 May 29;6:384. doi: 10.3389/fpls.2015.00384 (PMC4448004; doi:10.3389/fpls.2015.00384)
Supplement: Supplementary file 1 [file Table1.DOCX]

Table 1. Composition of culture media used in onion gynogenesis with protocol A (Jakše and Bohanec, 2003) and protocol B (Michalik *et al*., 2000), somatic embryogenesis (Luthar and Bohanec, 1999), and elongation medium for plant development (Jakše and Bohanec, 2003).

| Compounds | Gynogenesis | | | | | Somatic Embryogenesis | |  | Elongation |
| --- | --- | --- | --- | --- | --- | --- | --- | --- | --- |
|  | Protocol A |  | Protocol B | |  |  |  |  |  |
|  | Induction |  | Induction (A_1_) | Regeneration (R_1_) |  | Induction (I) | Regeneration (R_2_) |  |  |
| Macronutrients | BDS |  | B5 | BDS |  | BDS | BDS |  | BDS (x1/2) |
| Micronutrients | BDS |  | B5 | BDS |  | BDS | BDS |  | BDS (x1/2) |
| *Vitamins, amino acids and other nutrient elements(mg/l)* | | | | |  |  |  |  |  |
| Fe | 40 |  | 40 | 40 |  | 40 | 40 |  | 40 |
| Thiamine | 10 |  | 2 | 2 |  | 10 | 10 |  | 10 |
| Pyridoxine | 1 |  | 1 | 1 |  | 1 | 1 |  | 1 |
| Nicotinic acid | 1 |  | 1 | 1 |  | 1 | 1 |  | 1 |
| Ca panthotenate |  |  | 1 | 1 |  |  |  |  |  |
| Glicine |  |  | 2 |  |  |  |  |  |  |
| Folic acid |  |  | 1 |  |  |  |  |  |  |
| Biotin |  |  | 0.01 |  |  |  |  |  |  |
| Adenine |  |  |  | 10 |  |  |  |  |  |
| Myo-inositol | 500 |  | 100 | 500 |  | 500 | 500 |  | 250 |
| L- Proline | 200 |  |  | 200 |  | 200 | 200 |  | 100 |
| *Growth regulators (mg/l)* | | | |  |  |  |  |  |  |
| 2,4-D | 2 |  | 2 |  |  | 2 |  |  |  |
| BAP | 2 |  | 2 |  |  | 2 |  |  |  |
| 2iP |  |  |  | 2 |  |  |  |  |  |
| NAA |  |  |  | 1 |  |  |  |  |  |
| Thidiazuron |  |  |  |  |  |  | 2 |  |  |
| *Other components (g/l)* | |  |  |  |  |  |  |  |  |
| Sucrose | 100 |  | 100 | 100 |  | 100 | 50 |  |  |
| Glucose |  |  |  |  |  |  |  |  | 15 |
| Agar Daishin | 7 |  | 7 | 7 |  |  |  |  | 7 |
| Phytagel |  |  |  |  |  | 2.5 | 2.5 |  |  |
| pH | 6.0 |  | 5.8 | 5.8 |  | 6.0 | 6.0 |  | 6.0 |
